# IoT Wearable Sensors and Devices in Elderly Care: A Literature Review

**DOI:** 10.3390/s20102826

**Published:** 2020-05-16

**Authors:** Thanos G. Stavropoulos, Asterios Papastergiou, Lampros Mpaltadoros, Spiros Nikolopoulos, Ioannis Kompatsiaris

**Affiliations:** Centre for Research & Technology Hellas, Information Technologies Institute, 6th Km Charilaou-Thermi, 57001 Thessaloniki, Greece; astepapa@iti.gr (A.P.); lamprosmpalt@iti.gr (L.M.); nikolopo@iti.gr (S.N.); ikom@iti.gr (I.K.)

**Keywords:** IoT, wearables, sensors, devices, elders, old age, AAL, Alzheimer’s, dementia

## Abstract

The increasing ageing global population is causing an upsurge in ailments related to old age, primarily dementia and Alzheimer’s disease, frailty, Parkinson’s, and cardiovascular disease, but also a general need for general eldercare as well as active and healthy ageing. In turn, there is a need for constant monitoring and assistance, intervention, and support, causing a considerable financial and human burden on individuals and their caregivers. Interconnected sensing technology, such as IoT wearables and devices, present a promising solution for objective, reliable, and remote monitoring, assessment, and support through ambient assisted living. This paper presents a review of such solutions including both earlier review studies and individual case studies, rapidly evolving in the last decade. In doing so, it examines and categorizes them according to common aspects of interest such as health focus, from specific ailments to general eldercare; IoT technologies, from wearables to smart home sensors; aims, from assessment to fall detection and indoor positioning to intervention; and experimental evaluation participants duration and outcome measures, from acceptability to accuracy. Statistics drawn from this categorization aim to outline the current state-of-the-art, as well as trends and effective practices for the future of effective, accessible, and acceptable eldercare with technology.

## 1. Introduction

The world’s population is increasingly aging [1]. People aged above 65 years old amount to 702.9 million in 2019, projected to reach 1548.9 million in 2050, marking a 120% increase. Likewise, people aged above 80 years old amount to 53.9 million in 2019, projected to reach 109.1 million in 2050, marking a 102.6% increase. This is causing a similar shift in terms of lifestyle and, naturally, healthcare needs, towards ailments associated primarily (but not exclusively) with elders. The most prominent of those is dementia, in its early and progressed forms, from mild cognitive impairment (MCI) to Alzheimer’s disease (AD), a neurodegenerative disorder with several cognitive and functional limitations. People living with dementia globally amount to 50 million in 2019 and are expected to triple to 150 million by 2050 [2]. Most of them (around 80%) are elders over 75 [3]. Seventy percent of them cannot live independently without assistance from a caregiver [4]. Yet, there is no solution for reversing cognitive-related challenges in the patient population. Holistic and objective information to clinicians about patient health status can drive tailored interventions to alleviate the ailments and slow down the progression of the disease. However, this imposes a huge burden to informal caregivers and healthcare professionals. The same burden is imposed by physical frailty, inability to conduct daily activities independently, and cardiovascular disease (CVD) associated with old age [5].

In this light, the Internet of Things (IoT) is a promising solution to offer continuous, objective, and holistic monitoring, alleviating the burden of human caregiver effort and supporting clinical decision making. IoT is a generally new concept, providing the possibility of healthcare monitoring with the use of wearable devices. IoT can be defined as a network of physical objects with embedded technology for sensing, interacting with the environment, and offering autonomous communication. Wearable devices with sensors are a popular application of IoT that attracted much attention in the last decade, to the point of affordable fitness applications in the retail market. Such wristbands or smartwatches can monitor an individual’s activities through day and night, without much interruption and discomfort [6] The range of wearables is widening from watches to smart textiles, electronics in clothes, belt-worn PCs, and smart glasses. Analytics and artificial intelligence (AI) techniques are often coupled with wearables and IoT to extract intelligence, patterns, trends, user profiles, outliers for deeper assessment, and care [7].

As an emerging area of research, assistive technologies for elders present much room for a comprehensive, systematic review to identify current practices and future trends and opportunities. Current literature reviews have surveyed a limited number of papers and have yet to produce wider set of indexes, aspects, and features that such technologies offer for the care of dementia, as well as other elderly-associated ailments such as frailty and CVD [8].

This paper presents such a systematic literature review of IoT sensors and devices in elderly care. It targets both existing review studies and case studies, and identifies common parameters-both technological, such as the type of devices, as well as clinical, such as their healthcare focus from AD to frailty and CVD, clinical trial duration, and cohort size. Of the immense amount of work in technology for elderly care, recent individual studies in the past decade (2010 to 2019) were identified. From those, review studies are examined first, presenting their aspects in terms of health focus, device types, and criteria examined. Then, the review goes into deeper detail of individual case studies, identified through citations of the aforementioned earlier reviews and a wider literature search. It presents common aspects among them, such as health focus, aims, device types, experimental evaluation duration, participants (cohort), and outcome measures. Through the common aspects, statistics are drawn outlining current state-of-the-art in the field, as well as future trends and effective practices to invest in the future of eldercare with technology.

The paper is structured as follows. Section 2 presents earlier review studies and their aspects. Section 3 reviews individual case studies, examining common aspects such as health focus, IoT technology, aim, and evaluation. Section 3 presents outcomes and statistics from the review. Section 5 reports limitations and challenges, and Section 6 presents conclusions.

## 2. Related Review Studies in IoT Wearable Sensors and Devices for Eldercare

**Figure 1:**
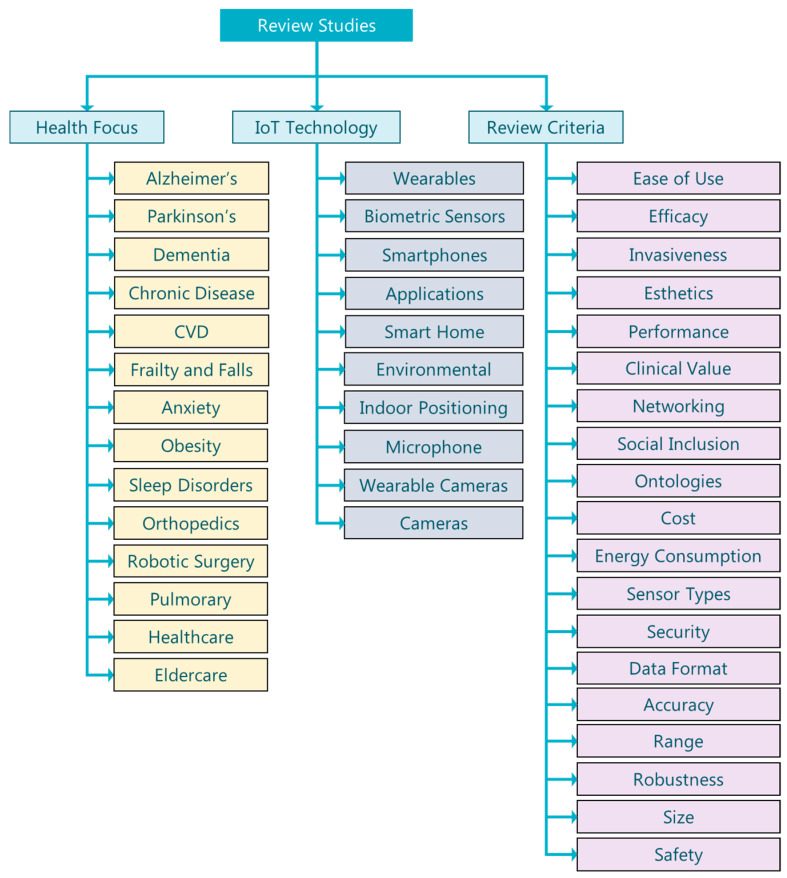
Review study classification taxonomy. IoT, Internet of Things; CVD, cardiovascular disease.

In this section, we present related work in the form of literature reviews in the area of IoT device and sensor technology for the care of elders. The work in this area is segmented into several categories in multiple aspects. We identify those aspects to be mainly three aspects: “health focus”, “IoT technology”, and “review criteria”, represented graphically in Figure 1.

Table 1 shows an evaluation of review studies according to those aspects, always following a distinct set of values for each aspect. The following subsections present each aspect and how current review studies perform in them.

### 2.1. Health Focus

The health focus aspect represents the healthcare-related, medical, or clinical aims the studies examine. Some of them are ailments and disease types or general “healthcare” and “eldercare”. In the former category, we find “dementia” in all its forms and pre-stages, including subjective cognitive impairment (SCI) and mild cognitive impairment (MCI) [27], and “Alzheimer’s” (AD) as a special case of sever dementia, “Parkinson’s” disease (PD), “CVD”, “frailty and falls”, “orthopedics”, “robotic surgery”, “pulmonary” disease, “anxiety”, “obesity”, “sleep disorders”, or “chronic disease” in general. To begin with, eldercare refers to care of elders with no specific ailment in mind, but rather monitoring and maintaining an active and healthy lifestyle in old age, prolonging independent living (so-called living in place), also referred to as active and healthy ageing (AHA) and often achieved through ambient, smart home, unobtrusive assistive technology-the so-called ambient assisted living (AAL) [23,24,25]. Beyond general healthcare and into specific ailments, AD and dementia are the most prominent of them. Although AD is a subtype, or a severe, progressed stage of dementia, some studies focus especially on that, such as Surendran et al. [15]. Others refer to the general spectra of dementia and AD alike, such as [8,10]. Dementia is sometimes presented as a sole health focus [14] or examined along other chronic disease in general [4] or frailty and falls [16]. PD is a popular health focus [9] combined with AD and dementia, and even CVD [11,12]. The study in [26] considers general eldercare, that is, prolonged independent living, as well as falls related to frailty.

In the less popular application areas, a lot of wearable devices can detect parameters such as blood pressure [20] and oxygen levels in blood [17], and thus constitute a very useful tool in the hands of persons with diabetes in CVD [19], arthritis, and orthopedics [13]. These devices measure sleep and asthma, related to anxiety and sleep disorders [18], as well as general eldercare [22]. Some reviews consider general healthcare provision, which includes eldercare [6,21], and focus on the more technical aspects such as encryption and data safety [28], examined in the next sections.

### 2.2. IoT Technology

The IoT technology aspect considers the various IoT wearable sensors and devices found in earlier review studies, mainly categorized in “wearables”, “smartphones”, “robotics”, “smart home”, “environmental sensors”, “indoor positioning”, “biometric sensors”, (fixed) “cameras”, “wearable cameras”, “microphone”, and “applications”. While all categories refer to specific hardware, the latter refers to any type of software and AI algorithm on local PCs or the cloud, which does not require a hardware IoT component of the former categories.

The study in [26] considers five types of devices: PIR motion sensors, body-worn sensors, pressure sensors, video monitoring, and sound recognition. Our review generalizes further to include more device types that are not considered there; for example, PIR motion sensors and pressure sensors are included in “smart home” sensors along with other possible types such as door-widow sensors, appliance and object usage sensors, and so on. Body-worn sensors are essentially “wearables”, and video monitoring and sound recognition are mapped to “cameras” and “microphones”, respectively, in our review.

To begin with, “wearables” are dominant in the literature, owing to their increasing popularity and affordability. Cedillo et al. [22] selected the most relevant devices to an AAL context, combining “wearables” and “applications” that contribute to the wellbeing of elders. Piwek et al. [18] includes various types of wearables, such as headbands, sociometric badges, camera clips, smartwatches, and sensors embedded in clothing, while Haghi et al. [6] deal with nine different motion trackers and four commercially available wrist-worn devices in the market for vital signs measurement, that is, FitBit, Jawbone, Withings, and Misfit. Another study [24] complements this list of commercial wrist-worn devices with Apple iWatch, Samsung Gear S2, Pebble Time, UP4 by Jawbone, Empatica, and Fitbit Flex, among others, through head-mounted devices and other accessories, such as smart jewelry, e-textiles, skin patches, and even an e-tattoo. In addition to both commercial devices and research prototypes, this review also examines pertaining potential security threats and confidentiality issues. Surendran et al. [15] explores smart wearable locator band, smart socks, the CleverCare Smart watch, iTraq, MedicAlert Safely Home, PocketFinder, Trax, and wearable cameras.

Biometric sensors are a special type of wearable or non-wearable devices that are used for both continuous and on-demand measurement of physiological and medical data. While they are often applied to security, for example, through fingerprint scanning, they are also used in healthcare, for example, measuring body temperature, electrocardiogram (ECG), pulse oxygen saturation, blood pressure, blood glucose, and so on [29]. Patel et al. [11] examines both smart home sensors for in-house positioning and microphones to record audio and voice, as well as a wide range of biometric sensors for glucose, pH, and O2 measurements. Another study [9] deals with what IoT offers to the neurological aspects of health disorders, examining devices that can be classified as both “wearables” and ”biometric sensors”, such as the Basis Health Tracker, Misfit Shine, Fitbit Flex, Withings Pulse O2, Actiwatch Spectrum, FitBit, Empatica 4, Bittium Faros, and PhysioCam. It also mentions an in-ear sensor for EEG (electroencephalogram). The study in [19] examines four wearables from a medical point of view, namely, Myo, Zyo patch, MyDario, and SleepBot. Along those lines, the study of [20] examines wearables and biometric sensors for diabetes, heart monitoring, and pulmonary disease, including radio-frequency identification (RFID) and wireless sensor networks (WSN) parameters.

Smart home devices are usually ambient and inobtrusive in an AAL context. A study from Wang [17] reviews indoor positioning systems, emphasizing on human activity recognition, as well as biometric sensors (vital sign monitoring, blood pressure, and glucose). Blackman et al. [25] consider three generations of AAL, gathering 64 studies, and consider parameters such as social support, interface, and health monitoring capabilities. They include wearables and smart home sensors (AiperCare, Aladdin, bed occupancy sensor, and so on), as well as environmental sensors such as gas detectors. The review in [14] deals with most types of “smart home” ambient sensors, “wearables” and “wearable cameras”, e-textiles, and “indoor positioning” systems, especially oriented around AAL projects.

Fall detection, prevention, and risk assessment mainly involve wrist-worn sensors, RFID sensors, and a footwear, as reviewed in Baig et al. [23]. The researchers in [16] also review AAL platforms, with wearables and smart home sensors to enable multimodal fall detection. Related to that, Ienca et al. [8] cover a wide area of intelligent assistive technologies around mobility and rehabilitation aid.

### 2.3. Review Criteria

Review criteria are used in earlier studies to evaluate, examine, and classify solutions offered in the surveyed case studies. In this paper, they are classified as follows: “sensor types”, “data format”, “ease of use”, “efficacy”, “invasiveness”, esthetics”, “performance”, “networking”, “ontologies”, “safety”, “security”, “robustness”, “cost”, “energy consumption”, “accuracy”, “range”, “social inclusion”, and “clinical value”.

The first set of criteria considers IoT technology and infrastructure parameters such as sensor types, networking architecture, and communication protocols. Salih et al. [13] refer to sensor types in wireless sensor networks (WSNs) for various sensing modalities, while also reviewing algorithms and intelligence applications of artificial neural networks (ANNs), activity prediction, and decision making. Similarly, the study in [10] reviews sensor characteristics, existing AAL platforms that stem from collaborative projects, and activity recognition systems.

Sensor types and networking are also considered in Banaee et al. [12], who additionally examine data mining from wearables to provide valuable information. Li et al. [4] review smart home and health care solutions, while emphasizing healthcare, rehabilitation, and AAL infrastructure with mobility assistance applications of robotic service platforms, multi-agent systems, and other human machine interfaces. Lee et al. [21] explores the field of sustainable wearables, while Surendran et al. [15] explores the networking and accuracy of several wearables and cameras. The study in [26] considers the types of sensors in five categories and especially their efficacy in various short studies (non-longitudinal).

Networking also entails communications and, many times, the data acquisition techniques. The study in [10] considers the various types of communication between devices and gateways, usually smartphones or PCs. Transmitter and receiver size is mentioned in [11], where a smaller size may be beneficial to weight, but reduces performance in transmission bandwidth. Banaee et al. [12] consider data acquisition for training algorithms as a criterion. Moreover, Li et al. [4] examine communications between devices as well as software agents in multi-agent systems. Some other technological aspects taken into consideration in some reviews are data format and data rate [14]; networks, data sets, models, and ANNs [12], update rate, data output, and algorithms [17]; or CPU, connectivity, memory, GPS, RAM, display, design, and communication capabilities [24].

When considering infrastructure, performance, and sustainability, energy consumption is also considered [11,21]. This plays an important role in portables and wearables as it attributes to comfort, but increases size [20]. Referring to elders, long battery life-and thus low power consumption-is all the more critical [23], as they are not familiar with consistently charging their devices. Thus, battery issues need to be minimal, or ideally, not exist at all [24]. Battery size and comfort usually relate to cost, but wearables become increasingly more portable, long-lasting, and affordable in retail, but maybe less durable and accurate [10].

Regarding security, Azzawi et al. [28] review data acquisition, processing, and analyzing parameters of body wearable sensors. They identify the need for secure infrastructure, in terms of new authentication mechanisms tailored to IoT devices. Network architecture [4] and strong authentication and encryption [28] can be different aspects of each device. Data security, in general, is a very important parameter [13]-the study of [18] and other reviews categorize devices with this criterion quite often.

Important criteria when it comes to healthcare and the elderly revolve around ease of use, size, and invasiveness, which ultimately shape the acceptance factors of the technology. This criterion is a fundamental concern for several studies [9,10]. The latter study also considers “easy installation”, which is an important parameter as well. More aspects relate to ease of use, such as compactness in [11,21], connectivity and easy device management [19], weight [6], and whether the user needs to operate a device or not [17]. Piwek et al. [18] also include the criterion of “behavioral effect”, which examines whether a device alters the user’s behavior in their everyday life. Blackman et al. [25] review the importance of a specialized user interface, as every user has different technological competence and literacy. Moreover, an emergency button is examined as a useful functionality of wearables for elders. Finally, esthetics also play a role in elderly users, many times with respect to stigma [9].

Some studies go into clinical validation, such as Ienca et al. [8], which takes into consideration the evidence of clinical validation for each device and the direct applicability to their health focus. Moreover, in [9], the wearables’ efficacy in the current health focus was reviewed. Lastly, two important criteria are safety [21,25] and daily tasks evaluation-two aspects of elders’ everyday life, which is the primary focus.

## 3. Review of Case Studies

This section presents a detailed review of case studies of IoT wearable sensors and devices for eldercare. In this paper, we use the general term “case study” to refer to any published study related to the topic, of any type. Some of them might be observational, interventional, or usability studies. These can be discriminated in this review through their “aims”, which are usually “monitoring” and “intervention” for observational and interventional studies, respectively. Usability studies can be discriminated through their evaluation outcome measures, which are mostly “acceptance”, “user satisfaction”, and “feedback”. All those aspects and categories are explained below.

**Figure 2:**
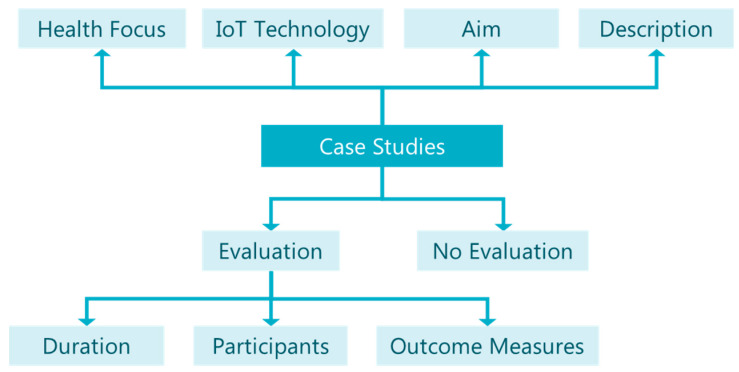
Case study classification taxonomy.

First, we present our proposed aspects and classification criteria of case studies, shown graphically in Figure 2. All studies share the same aspects, “health focus” and “IoT technology”, similar to review studies in the previous section, as well as more detailed information per-case, that is, “aim”, “description” and “evaluation”. Furthermore, we classify case studies to those with and those without an experiment for evaluation. Studies with evaluation are also examined for the aspects of “duration”, “participants”, and “outcome measures” related to it.

Table 2 presents what we have identified as recent and representative works according to the previous classification. The following subsection presents how the studies consider each aspect. Studies with an evaluation experiment are examined at the end as they entail even more aspects pertaining to evaluation duration, participants (cohort), and outcome measures.

### 3.1. Health Focus

Most case studies found present a device, or more, that measures different parameters of a disease. Alzheimer’s disease (AD) is the most common disease included in our study. There are some studies that focus on devices for the general elder population [31], dementia [37,39,49,51], Parkinson’s disease [41], and fall detection [35,36], or even combining some, or all, of the above mentioned [30,34,40,47].

### 3.2. IoT Technology

The devices presented here are mostly Wearables. Other types of sensors include smartphone Android apps [32]; all types of smart home ambient sensors [38], for example, a wireless doorbell system presented by Lancioni et al. [50]; rehab devices applications [53]; and so on. The various types follow those classified previously in related review studies.

### 3.3. Aim

This category is very general for the studies examined. Some of the reviews presented the aim of the detection of specific symptoms or behaviors arising from a person that has a known disease. Such review studies are [30,38,41,42,43]. The aim of [35,40,44,45,46,50] is prediction, which refers to predicting a disease of a healthy subject, via symptoms, repetitive behavior, and so on. When studying [30,34], it is easily outlined that there is an interest for emergency situations. Another focus of this category is monitoring [32,47]. These studies refer to a monitoring system for patients with a specific disease. Furthermore, another aim found in [36,39,51] is development. These reviews focus on developing an algorithm or a specific architecture for a system, so it measures specific characteristics. Two studies [31,37] focus on comparing the reviewed subjects, while two others’ [33,52] aim is tracking the patient, so the caregiver can be more comfortable or even the subject themself can be more independent. Finally, there are also aims such as biometric measurements [33], recall of some memories [48], patients’ improvement [49], and rehabilitation [53].

### 3.4. Description

Rodrigues et al. [30] used a smartphone and a smartwatch, which had simple interaction with the user, a fall alert, and an OK button to tap if the elder was well after falling or if he/she was lost and wandering. Ehrler & Lovis [31] present the advantages and disadvantages of smartwatches in the current study field, such as ubiquity, activity sensors, user adoption and safety, personalization, price, continuous medical surveillance, and appropriate ground to implement a platform with multiple services for elders, as well as considering disadvantages such as physical constraints like tiny screen size, small connectors, and limited power autonomy. Sharma and Kaur [32] developed an Android app that can access the disease’s symptoms data. Each user can find out if he suffers from the disease or not, as well as contact the doctor directly via messages and calls. They also propose a framework reducing communication cost.

A novel feature in the app’s language was presented by Aljehani et al. [33], who constructed an app for Apple smartwatch that supports the Arabic language. The app measures heart rate and locates the patient, who showed over 94% satisfaction using it. Bose et al. [34] constructed a sensor that measures heartbeat, acceleration, blood pressure, and body temperature. Its data is safely transferred to the remote control station via a developed wireless mesh network architecture. Some of its advantages are wireless signal detection, reliable data collection and transmission, low power, and efficient channel allocation. Karakaya et al. [35] used a smart watch (with an accelerometer and gyroscope) with a mobile app as a system to collect sensory data for the elderly people’s activities prediction. The app reads the outputs of the sensors in a smart watch continuously and uploads them to a web service, which initiates a classifier program to predict the activity and can communicate with the smart watch to warn or check the user condition. Finally, Khojasteh et al. [36] developed a dataset and an artificial neural network, using a smartwatch for data collection. Their method, however, needs to be validated with more datasets, specifically from real fall events. Some of its features are usability, ergonomic solutions to problems, users’ comfort, less communications, and more battery life.

### 3.5. Evaluation

The “evaluation” column in Table 2 categorizes the case studies found according to having tested the presented device with a study group. This section dives further into studies with evaluation and examines their pertaining aspects, namely, “study duration”, “participants” (study cohort size, demographics, health condition, and so on), and “outcome measures”, as shown on Table 3.

#### 3.5.1. Duration

Study durations can vary. Studies [41,45,50] are minute-long trials, while the studies in [40,44,46,49] last for a couple of hours. Longer studies lasts from a couple of days to a whole week [37,39,42,47]. Several studies may last from a couple of months or case studies [38,48,52,53], while only one study lasts more than a year [51]. Statistics of their distribution are presented further in Section 4.

#### 3.5.2. Participants

The demographics of participants in each study are one of their most characteristic aspects. Several studies involve small groups of ten participants [39,47,49,51] or less [48,50]. Fewer studies fall into the range of ten to twenty [38,42] and twenty to thirty [40,53]. Even less studies involve more participants, from thirty to seventy [43,45,52] and from seventy to a hundred [41,44,46]. Only one study involves more than a hundred participants, with 178 subjects [37].

Another demographic is the age and gender of the participants and the presence of caregivers in the study. Thorpe et al. [39] examine pairs of subjects (two female and three male) and caregivers. Ellis et al. [40] study 12 patients (five female) and 12 health subjects (four female). Weiss et al. [41] has 96 patients with a 22% percentage of female subjects in it. Costa et al. [44] studied 36 AD patients (24 females, 12 males) with a mean age of 76 ± 7 years and 36 healthy subjects (15 females-21 males), with a mean age of 70 ± 8 years. Zhou et al. [45] have a 43.3% percentage of female subjects in their trial, while Woodberry et al. [48] deal with four female and two male subjects. Lancioni et al. [50] had six subjects and two studies. The first study had three participants from 75 to 89 years old and the second study had the same three plus one more, whose age is 71 years old. Jelcic et al. [53] examined 27 patients divided into three groups: seven of them followed the LSS-tele treatment, 10 of them followed the LSS-standard direct intervention, and 10 were the control condition. Lastly, Algase et al. [37] studied 178 patients, of which 75.3% are females.

Regarding drop-outs and attrition, unfortunately, not all papers clearly mention them nor the underlying reasons. One study does mention drop-outs due to adherence problems (*n* = 2) and technical problems (*n* = 1) [42]. The study in [40] mentions data loss, owing to connectivity issues; faults in file transfer; and equipment failure, resulting in dropping data of six participants from the study. However, those cannot be classified as drop-outs owing to the participant choice or circumstance. As a result, drop-outs cannot always be interpreted as adherence problems, but also technical ones, and even so, are not always mentioned. Therefore, they are left out of the survey in order to avoid misleading the reader.

#### 3.5.3. Outcome Measures

In accordance to evaluation aims, the outcome measures aim to assess mainly parameters of comfort or efficacy and effectiveness to either assess a condition, or to improve it, through monitoring and intervention. This section provides details on each evaluation and reports on their results.

Algase et al. [37] tested the four wearables Actillume, StepWatch, Step Sensor, and TriTrac-R3D. While Step Sensor was the staff’s preferred device, its performance was least acceptable for research purposes. StepWatch and Actillume were able to yield the largest amount of meaningful data. There were benefits such as appearance, comfort, ease of application and cleaning, location, safety, size, and weight. Hao et al. [38] applied in-house IoT sensors and targeted excessive active levels, abnormal sleeping patterns, and repetitive behavior, all indicating potential AD. Limitations faced were sensor quality, assumptions, and data combination with qualitative information. Thorpe et al. [39] tested a smartwatch and a mobile phone with various applications, and there was promise for user adoption overall, with scheduling as most successful and navigation as least successful in terms of usability and usefulness. Some of their recommendations for future work are using the smartwatch as output only, personalizing the solution to users’ individual needs, and making it as familiar to them as possible.

Moreover, Ellis et al. [40] examine gait with the iPod Touch in combination with two foot sensors and a mobile app, and found that, relatively to healthy (HE) subjects, patients with Parkinson’s disease (PD) walked with slower and took shorter steps, as well as increased step time and step length variability. Weiss et al. [41] issue a small, light-weight sensor attached to a Velcro elastic belt on the patient’s lower back to examine strategies that older adults take when they turn to sit. During testing without medication, about two-thirds of the participants performed the turn using the overlapping-strategy. Patients with PD were almost twice as likely to choose the overlapping strategy (part of the turning and sitting take place concurrently, in an overlapping manner) compared with the distinct strategy (turning is first completed and only then sitting begins). A single wearable is presented by Mc Ardle et al. [42], who demonstrate that gait could be a useful clinical biomarker to prevent dementia, as changes can occur up to 12 years prior to diagnosis of cognitive impairment. They found that people with mild AD walked more slowly and were more asymmetrical with impaired variability and postural control of gait compared with this reference control group. Silva et al. [43] examined three cognitive training groups, stating that the one with the wearable camera had significantly reduced depressive symptoms, and highlight that SenseCam is useful to stimulate not only cognitive function, but also overall function (affective, functional), even in a neurodegenerative condition such as AD.

In the meantime, Woodberry et al. [48] also use SenseCam and view images to patients and tested in parallel groups with diaries. SenseCam outperformed the diary method and showed improvement over time. The patients remembered more images from the SenseCam trial. Costa et al. [44] examined a triaxial accelerometer and gyroscope. Participants were exposed to seven increasingly difficult postural tasks and found high intercorrelation between the different proposed kinematic variables and substantial overlap between healthy subjects and AD patients. Zhou et al. [45] present a wearable sensor (triaxial accelerometer, gyroscope, and magnetometer) combined with a human-machine interface. Three tests between the subject and the PC were held and all subjects were able to complete all tests without any support from the study administrator. None of the participants were stopped or overtaxed during the test, indicating its feasibility for older adults, including those with MCI and dementia. Number-letter is the most sensitive test to identify motor-cognitive impairment among older adults. Excellent test-retest reliability was achieved, when alteration between numbers and letters was used. Hsu et al. [46] dealt with an inertial-sensor-based wearable device (a triaxial accelerometer and two gyroscopes) mounted on participants’ feet and waist. The participants were demanded to walk along two straight lines of 40 m, one for single-task walking and the other for dual-task walking, and another trial had eight balance ability tests. For the dual-task test, the AD group differed significantly from the HC group on number of strides, walking time, stride length, stride speed, stance time, stance period, swing period, CV of stand period, and CV of swing period under the dual-task condition. In single task walking, no significant differences in gait were observed. This indicated that the countdown motion is related to the cognitive function and attention, so the AD patients performed worse in those gait parameters than the HCs in dual-task walking. For the balance test, AD patients presented larger average sway speed in all of the rest parameters.

Finally, Abbate et al. [47] developed a four-component-system with two wearable sensors (waist and head), an in-house sensor, and a camera. The waist sensor achieved higher usability than the head sensor, so there had to be modifications to the latter. Leuty et al. [49] used ePAD (Engaging Platform for Art Development) in a small trial to engage persons with dementia in creative art occupations and users were highly satisfied. Lancioni et al. [38] developed a wireless doorbell system and examined two aspects: two activities requiring 20 steps by the patients, and then to reach five rooms to deliver some material. The results were improved after the intervention, but different outcomes may result from the small patient groups and from characteristics of the patients and/or of the travel routes. Aloulou et al. [51] developed a set of sensors and devices controlled by a software platform. The study had three phases, of which, during the first two, the interaction is only with the caregivers. They found that the majority of the patients’ unsupervised time was spent in their bedroom or washroom. Pot et al. [52] developed a tracking device combining GPS and GPRS, track and trace function, and telephone contact. Both carers and patients were less worried when being alone, were more often outside independently, and received more freedom from their caregivers. Jelcic et al. [53] developed a rehab protocol based on two applications run on two PCs (via Skype) that contained lexical tasks aimed at enhancing semantic verbal processing, and found that the use of telecommunication technologies could have influenced the profile of cognitive changes after rehabilitation.

## 4. Results and Statistics

**Figure 3:**
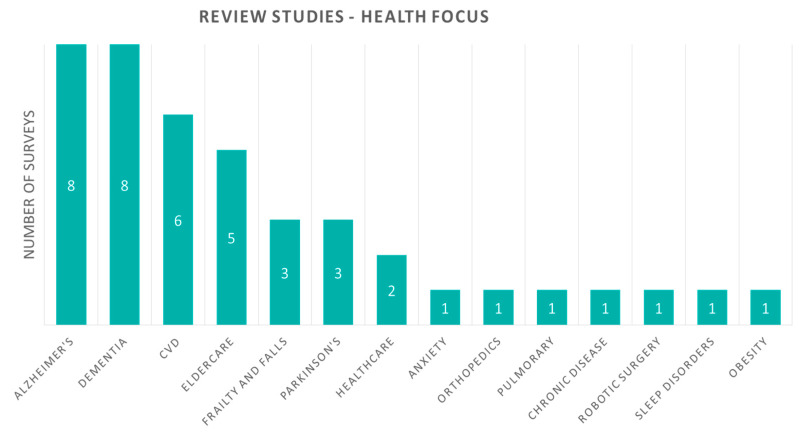
Review studies according to their health focus. CVD, cardiovascular disease.

In this section, the review outcomes are aggregated and presented visually. Starting with the review/survey studies utilized in this publication, Figure 3 presents graphically their health focus in categories. Alzheimer’s and dementia are the most reviewed, with eight studies each. Moreover, some of the studies reviewed combinations and not only a single health focus. Review studies referring to Alzheimer’s disease combine with Parkinson’s disease and dementia, while the latter combines with Alzheimer, cardiovascular diseases, and fall detection. Cardiovascular disease is the next most reviewed, with six studies (one of them combining with Fall Detection), and eldercare with five studies. Moreover, frailty and falls and Parkinson’s disease are represented in three studies each.

**Figure 4:**
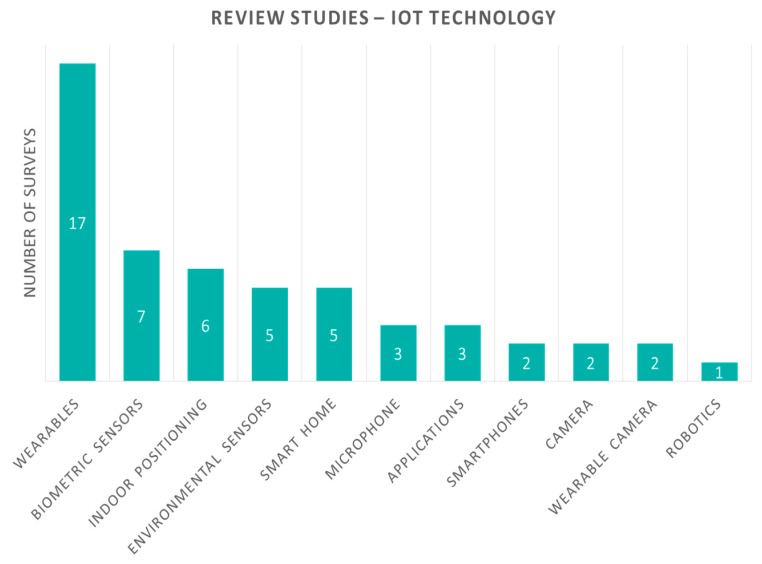
Review studies according to Internet of Things (IoT) technology devices are presented.

Review studies are also classified according to the IoT technology devices they are demonstrating (Figure 4). Wearables are reviewed in the majority of review studies, sixteen in number, which identifies their popularity in this scientific field. Biometric devices measure parameters such as blood glucose, blood oxygen, and so on, and are reviewed in six studies. Environmental sensors and indoor positioning sensors are reviewed in five studies each. Smart home and smartphone devices are reviewed in three studies each. Finally, camera, wearable camera, and microphone are featured in one study each.

**Figure 5:**
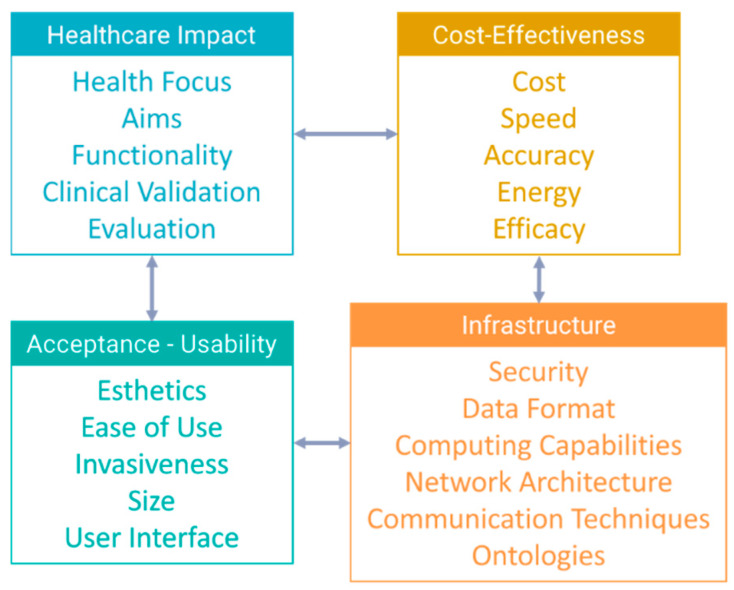
Categories of criteria examined in review studies.

Overall, another classification extracted from all criteria examined in past review studies is shown on (Figure 5). Four larger categories can be discriminated: (1) criteria affecting the amount of impact in a patient’s health, which can be health focus, aims, system functionality for the patient, clinical validation, and evaluation outcomes; (2) acceptance and usability parameters, which include esthetics, ease of use, invasiveness, and size of the devices, as well the system’s user interface; (3) cost-effectiveness, which regards accuracy, energy consumption, efficacy, cost, and speed parameters (latency, bandwidth, and so on); and (4) infrastructure, related to security and transport protocols, data models and ontologies, computing capabilities, and network architecture. Categories of parameters affect one another, for example, more capable infrastructure relates to cost-effectiveness or more acceptance and usability owing to decreased delay.

**Figure 6:**
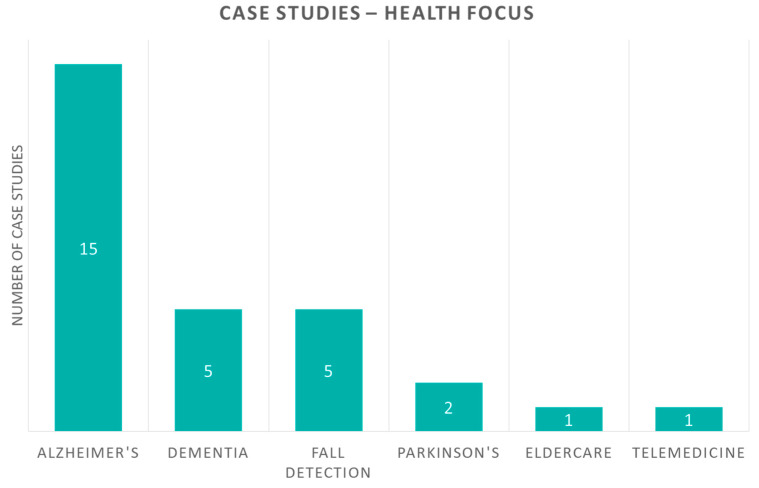
Case study papers according to their health focus.

Case studies are also separated according to their health focus. In Figure 6, the allocation of studies is presented. Alzheimer’s disease is once again the leading health focus with fifteen studies. Two of them are combined with fall detection and one with dementia. Dementia and fall detection are represented in five studies each. Finally, Parkinson’s disease appears in two studies, and the general categories of eldercare and telemedicine in one study each.

**Figure 7:**
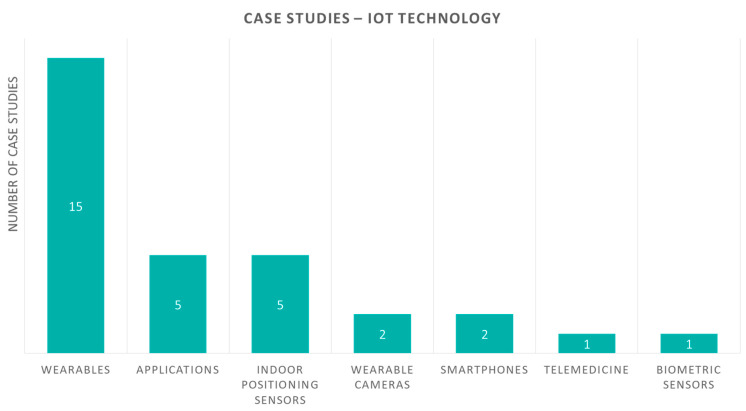
Case study papers according to the IoT devices used.

Another criterion is the IoT technology devices presented in each case study, which is demonstrated in Figure 7. The category of wearables, such as in the review studies, is represented by the majority of studies, fifteen in number. Moreover, in this category, the combination of wearables and applications is represented in five studies. Indoor positioning sensors are represented in five studies, and wearable cameras, smartphones and telemedicine in two studies each. Finally, biometric sensors and telemedicine are represented in one study each.

**Figure 8:**
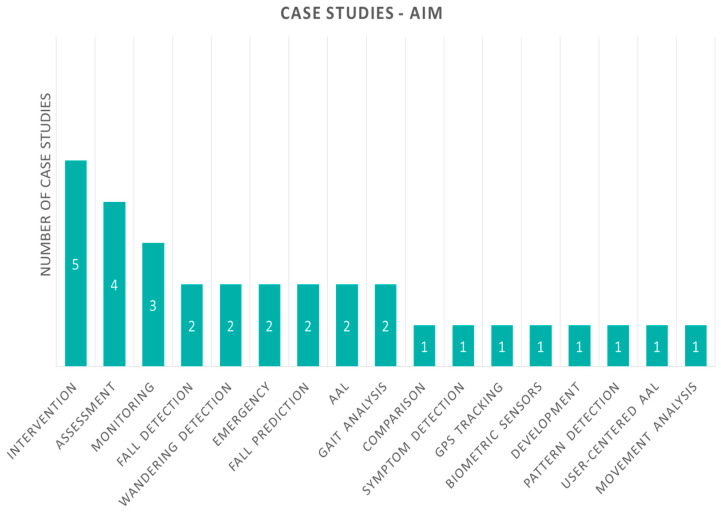
Case study papers according to their aim. AAL, ambient assisted living.

Studies classified by different “aims” are shown in Figure 8. There are five studies aiming for intervention, assessment comes second with four case studies, and monitoring third with three. Aims that were represented in two of the case studies each were fall detection, wandering detection, emergency, fall prediction, and AAL. The rest of the aims identified appear in one case study each and include comparison, symptom detection, GPS tracking, biometric sensors, development, pattern detection, user-centered AAL, gait analysis, and movement analysis.

**Figure 9:**
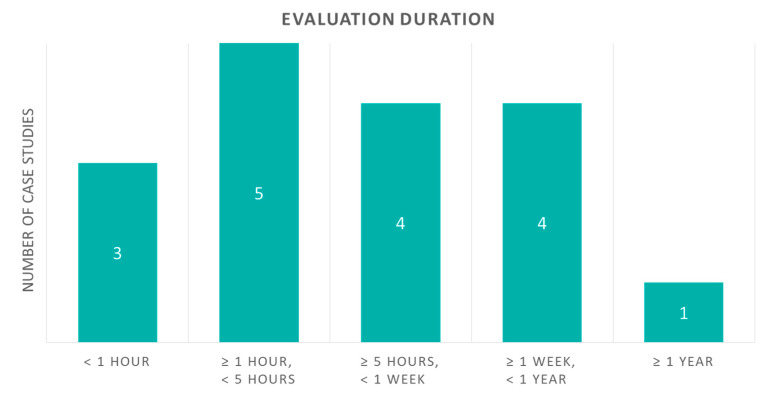
Case studies with evaluation according to their duration.

When considering case studies involving patients (i.e., studies with an evaluation), there are differences in their duration. It can be from some minutes to several months, as shown in Figure 9. Three studies examined last less than one hour; four of them last for some hours less than five; four of them for some days less than one week; four of them for several weeks or months, but less than a year; and only one study lasted for more than one year. The results can be explained from the fact that many studies need baseline periods, so is it sometimes inevitable to have a trial last for less than a week and so it might come to last for several months. On the contrary, there are some studies with very short trial periods, such as short gait examinations, that last from several minutes each or a couple to some hours long.

**Figure 10:**
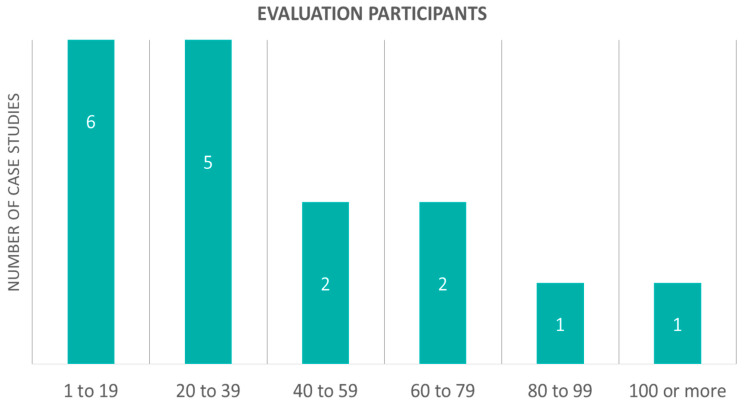
Case studies with evaluation according to their participant number.

Remaining in the field of clinical trials involving patients, there is another aspect that is worth considering. The number of persons included in each evaluation is a very important parameter and accordingly classifies the trial (Figure 10). The results suggest that a trial usually involves less than forty participants. More specifically, six studies involved 1 to 19 participants and five studies involved 20 to 39. Moreover, two studies involved 40 to 59 participants and the same number of studies involved 60 to 79 participants. As the number of participants increases, fewer studies are addressed. Additionally, a case study with more patients has a baseline period before and after the trial, so it is outlined that it will last longer. Consequently, one study involved 80 to 99 participants and, finally, more than 100 participants were involved in only one study.

**Figure 11:**
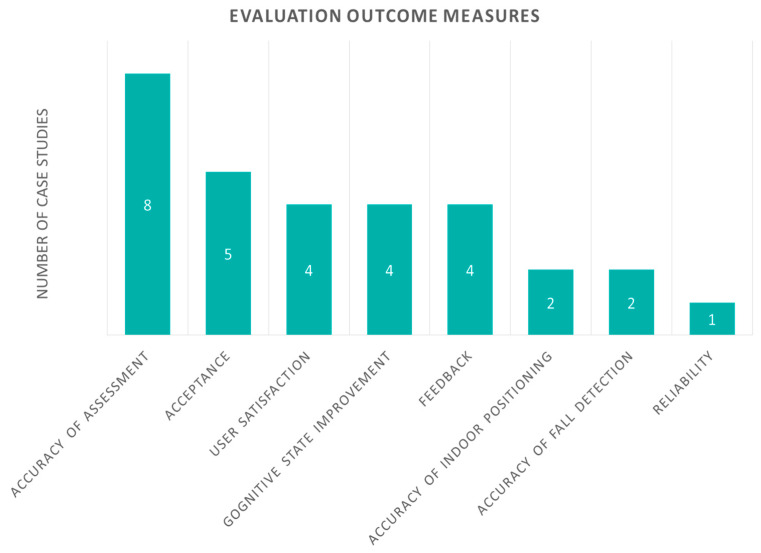
Case studies with evaluation according to their outcome measures.

Finally, outcome measures are featuring important aspects of the evaluation and are shown in Figure 11. The most common outcome measure categories, with six studies, are patients’ feedback and recognition of mental state. How patients value these studies is very important, and the researchers aim for patient approval and high quality results, considering all the parameters of the trial.

Most of the studies (eight) measure accuracy of assessment. Acceptance was evaluated in five and user satisfaction and cognitive state improvement were addressed in four studies each. Considering user acceptance, for some technologies, patients recognized that it was positively affecting their lives, but they were not comfortable enough to accept them. Patient feedback results also appear in four studies and no negative feedback was identified in general. Accuracy of indoor positioning and fall detection was demonstrated in two studies and reliability was analyzed in one study.

## 5. Limitations and Challenges

Both market penetration and literature research prototypes of IoT wearable sensors and devices have grown a lot over the past decade, with applications in many aspects of lifestyle and healthcare, including elderly demographics. A challenge that still remains is their acceptance, apparent as a factor in many studies. Acceptance entails ease-of-use of both hardware and software user interfaces, comfortability, size, weight, and battery life/energy consumption parameters. An optimal balance between such comfort parameters, usually met in lifestyle application of retail products, and performance, accuracy, and higher suitability for biometric applications in healthcare, is needed.

Another limitation is the lack of interoperability and a common platform. Many studies have presented segmented AAL projects in the area. Moreover, most studies integrate sensors and implement their own data acquisition techniques. Data interoperability post-acquisition is also limited across studies.

Finally, security, privacy, and ethics are a remaining concern. While standard secure storage and authentication techniques exist and are implemented in most systems, the sector could benefit from IoT specific frameworks for more efficient or autonomous authentication of devices. Privacy and ethics are also managed in an ad hoc manner per study, and could benefit from common frameworks established across vendors and organizations.

## 6. Conclusions and Future Work

Efficient, affordable, and accessible healthcare for the ever-growing demographics of elders and ailments pertinent to them is eminently needed. IoT wearable sensors and devices have been growing immensely in the last decade, penetrating the market and with both lifestyle and biomedical applications. The review presented in this paper explored literature in the sector exploring, identifying, and analysing common aspects among earlier reviews and individual case studies. Healthcare aspects range from chronic ailments, primarily AD and other forms of dementia, PD, frailty, and CVD, to general eldercare and AAL. IoT technologies are prominently wearables, as well as smart home sensors, cameras, microphones, and indoor and outdoor tracking. The major aims include assessment of cognitive state, frailty, and other conditions, as well as support, assistance, and prevention of falls. Besides assessment and monitoring, some studies constitute interventions themselves to improve the individual’s healthcare states. Open issues include common frameworks for interoperability, privacy, and security management tailored to IoT.

In accordance to that, future works in the form of novel studies or literature reviews could focus on the human aspects of IoT for eldercare. Such aspects entail technological hardware and software features and how they match human needs, particularly to elders and respective ailments. The balance between technological capabilities in terms of accuracy, performance, and modalities and human requirements for comfort, durability, and often esthetics is everchanging and could be investigated. Another aspect is the duration of the studies. With more advanced, durable, and comfortable technology, only made available recently, studies that explore longer-term effects and benefits could emerge. Constitution of Big Data from IoT wearables could soon emerge as predictive medical tools and digital biomarkers for elderly enabling care at home, as well as pharmaceutical treatment by accelerating and optimizing clinical trials.

## Figures and Tables

**Table 1:** Review studies and their aspects: health focus, Internet of Things (IoT) technology, and review criteria.

Review Study	Year	Health Focus	IoT Technology	Review Criteria
Talboom & Huentelman [9]	2018	Alzheimer’s,	Wearables	Ease of Use,
		Parkinson’s	Biometric Sensors	Efficacy,
				Invasiveness,
				Esthetics
Ienca et al. [8]	2017	Dementia,	Wearables, Smartphones,	Efficacy,
		Alzheimer’s	Applications,	Performance,
			Robotics	Clinical Value
Li et al. [4]	2015	Dementia,	Smart Home	Networking
		Chronic Disease		Social Inclusion,
				Ontologies
Al-Shaqi et al. [10]	2016	Dementia,	Biometric Sensors, Environmental Sensors,	Networking,
		Alzheimer’s	Indoor Positioning, Smart Home	Ease of Use,
				Cost,
				Efficacy
Patel et al. [11]	2012	Dementia,	Wearables,	Cost,
		Alzheimer’s,	Biometric Sensors,	Energy Consumption
		Parkinson’s,	Indoor Positioning,	
		CVD	Microphone	
Banaee et al. [12]	2013	Dementia,	Wearables	Sensor Types,
		Alzheimer’s,		Networking
		Parkinson’s,		
		CVD		
Salih et al. [13]	2013	Dementia,	Microphone,	Networking,
		Alzheimer’s,	Environmental Sensors,	Security
		CVD	Biometric Sensors,	
			Smart Home	
Rashidi & Mihailidis [14]	2013	Dementia	Wearables,	Sensor Types,
			Wearable Cameras,	Data Format
			Biometric Sensors,	
			Environmental Sensors,	
			Indoor Positioning	
Surendran et al. [15]	2018	Alzheimer’s	Wearables,	Networking,
			Wearable Cameras	Accuracy
Spasova & I. Iliev [16]	2014	Frailty and Falls,	Wearables,	Networking,
		Dementia,	Cameras,	Sensor Types,
		Alzheimer’s	Smart Home,	Efficacy
			Environmental Sensors,	
			Indoor Positioning	
Wang et al. [17]	2017	Frailty and Falls,	Indoor Positioning	Accuracy,
		CVD		Security,
				Networking,
				Range,
				Cost,
				Ease of Use
Piwek et al. [18]	2016	Anxiety,	Wearables, Smartphones, Applications	Robustness,
		Obesity,		Security
		Sleep Disorders		
Dimitrov [19]	2016	Orthopedics,	Wearables	Ease of Usage,
		Robotic Surgery,		Networking
		CVD		
Scarpato et al. [20]	2017	Pulmonary,	Wearables,	Energy Consumption,
		CVD	Biometric Sensors	Size
Haghi et al. [6]	2017	Healthcare	Wearables,	Cost,
			Biometric Sensors	Size,
				Energy Consumption
Lee et al. [21]	2016	Healthcare	Wearables	Robustness,
				Cost,
				Size,
				Energy Consumption
Cedillo et al. [22]	2018	Eldercare	Wearables,	Sensor Types,
			Applications	Networking
Baig et al. [23]	2019	Eldercare	Wearables	Ease of Use,
				Energy
Seneviratne et al. [24]	2017	Eldercare	Wearables	Energy Consumption
Blackman et al. [25]	2016	Eldercare	Wearables, Environmental Sensors,	Safety,
			Indoor Positioning	Ease of Use
Peetoom et al. [26]	2015	Eldercare,	Wearables,	Sensor Types,
		Frailty and Falls	Smart Home,	Efficacy
			Cameras,	
			Microphone	

**Table 2:** Review of case studies and their aspects. AD, Alzheimer’s disease; PD, Parkinson’s disease’ AAL, ambient assisted living.

Case Study	Year	Health Focus	IoT Technology	Aim	Description	Evaluation
Rodrigues et al. [30]	2018	Alzheimer’s, Fall Detection	Wearables, Smartphones	Fall Detection, Wandering Detection, Emergency	Fall and wandering detection for emergency alerts	-
Ehrler & Lovis [31]	2014	Eldercare	Wearables	Comparison	Smartwatches for elderly support	-
Sharma & Kaur [32]	2017	Alzheimer’s, Telemedicine	Smartphones, Applications	Monitoring, Symptom Detection	Android app to monitor AD symptoms and contact doctors	-
Aljehani et al. [33]	2018	Alzheimer’s	Wearables, Applications	GPS Tracking, Biometric Sensors	GPS and heart rate logging	-
Bose [34]	2013	Dementia, Alzheimer’s	Biometric Sensors	Emergency	Detect emergency and send alerts	-
Karakaya et al. [35]	2017	Fall Detection	Wearables, Applications	Fall Prediction	Predictive model for falls	-
Khojasteh et al. [36]	2018	Fall Detection	Wearables	Development	Fall detection from wrist-worn sensors	-
Algase et al. [37]	2018	Dementia	Wearables	Wandering Detection	Four devices for wandering detection	✓
Hao et al. [38]	2017	Alzheimer’s	Indoor Positioning Sensors	Pattern Detection	Detect indoor movement patterns of AD	✓
Thorpe et al. [39]	2016	Dementia	Wearables, Applications	User-centered AAL	User-centered approach to develop AAL	✓
Ellis et al. [40]	2015	Fall Detection, Parkinson’s	Wearables, Applications	GAIT Analysis	GAIT analysis from two devices	✓
Weiss et al. [41]	2019	Parkinson’s	Wearables	Movement Analysis	Movement analysis (turn and sit) for PD	✓
Mc Ardle et al. [42]	2018	Alzheimer’s	Wearables	GAIT Analysis	GAIT analysis, acceptability, and feasibility	✓
Silva et al. [43]	2017	Alzheimer’s	Wearable Cameras	Intervention	Camera intervention for improvement	✓
Costa et al. [44]	2016	Alzheimer’s	Wearables	Fall Prediction, Assessment	Fall prediction and AD assessment	✓
Zhou et al. [45]	2016	Alzheimer’s	Wearables	Assessment	Motor-cognitive assessment	✓
Hsu et al. [46]	2014	Alzheimer’s	Wearables	Assessment	Indicators for AD assessment	✓
Abbate et al. [47]	2014	Alzheimer’s, Fall Detection	Wearables, Indoor Positioning	Fall Detection, Monitoring	Long-term monitoring and fall detection in nursing homes	✓
Woodberry et al. [48]	2015	Alzheimer’s	Wearable Cameras	Intervention	External memory aid to promote recall of episodic memories	✓
Leuty et al. [49]	2013	Dementia	Wearables	Intervention	Promote engagement, art creation	✓
Lancioni et al. [50]	2013	Alzheimer’s	Indoor Positioning	AAL, Intervention	Indoor activity and travel support	✓
Aloulou et al. [51]	2013	Dementia	Indoor Positioning	AAL	AAL in nursing homes	✓
Pot et al. [52]	2012	Alzheimer’s	Indoor Positioning	Monitoring	GPS for indoor tracking	✓
Jelcic et al. [53]	2014	Alzheimer’s	Telemedicine	Assessment, Intervention	Lexical-semantic stimulation through Telecommunication	✓

**Table 3:** Review of case studies with evaluation and their aspects. MCI, mild cognitive impairment.

Case Study	Year	Study Duration	Participants	Outcome Measures
Algase et al. [37]	2018	1 Week	178 (mean age 85.3 y/o)	Acceptance, Accuracy of Wandering Detection
Hao et al. [38]	2017	6 Months	20	Accuracy of Assessment by Pattern Detection
Thorpe et al. [39]	2016	7 Days	10 (61–73 y/o)	Acceptance, Feedback
Ellis et al. [40]	2015	1–2 h	24: 12 PD & 12 HC (40–85 y/o)	Accuracy of Assessment by GAIT Analysis
Weiss et al. [41]	2019	Less than 1 h	96 PD	Accuracy of PD Assessment by Movement Analysis
Mc Ardle et al. [42]	2018	7 Days	20 (55–80 y/o)	Acceptance, Accuracy of Assessment by GAIT Analysis
Silva et al. [43]	2017	6-Week Trial, 6-Month Follow-up	51 AD (60–80 y/o)	Cognitive State Improvement through Intervention
Costa et al. [44]	2016	2–3 h	72: 36 AD (76 ± 7 y/o), 36 HC (70 ± 8 y/o)	Accuracy of Fall Detection and Assessment
Zhou et al. [45]	2016	5 Min Session	30: 11 HC, 10 aMCI, 9 AD (71–93 y/o)	Reliability, Accuracy of Motor-cognitive Assessment
Hsu et al. [46]	2014	A Few h	71: 21 AD & 50 HC	Accuracy of Assessment
Abbate et al. [47]	2014	2–4 Days	4 AD (75–92 y/o).	Acceptance, User Satisfaction, Accuracy of Fall Detection
Woodberry et al. [48]	2015	3.5 Months	6 (64–84 y/o)	User Satisfaction, Cognitive State Improvement through Intervention
Leuty et al. [49]	2013	Five 1-Hour Trials	6 (mean age 89.2 y/o)	User Satisfaction, Feedback
Lancioni et al. [50]	2013	Ten 1-Minute Trials	6 (75–89 y/o)	Cognitive State Improvement through Intervention
Aloulou et al. [51]	2013	14 Months	10: 8 AD, 2 Carers	Feedback, Accuracy of Indoor Positioning
Pot et al. [52]	2012	3 Months	56 Patient-Carer pairs	User Satisfaction, Acceptance, Feedback, Accuracy of Indoor Positioning
Jelcic et al. [53]	2014	3 Months	27	Cognitive State Improvement through Intervention, Accuracy of Assessment
